# Reply to comments on “Efficacy of modified FOLFOX6 chemotherapy for patients with unresectable pseudomyxoma peritonei”

**DOI:** 10.1007/s10147-021-01878-z

**Published:** 2021-02-06

**Authors:** Sakura Hiraide, Keigo Komine, Masanobu Takahashi, Chikashi Ishioka

**Affiliations:** 1 Department of Clinical Oncology, Tohoku University Graduate School of Medicine, 2-1, Seiryo-machi, Aoba-ku, Sendai, 980-8575 Miyagi Japan; 2 Department of Clinical Oncology, Institute of Development, Aging and Cancer, Tohoku University, Aoba-ku, Sendai, Miyagi Japan; 3 Department of Medical Oncology, Tohoku University Hospital, Aoba-ku, Sendai, Miyagi Japan

We thank Dr. Luo and Dr. Li for providing comments regarding our manuscript entitled “Efficacy of modified FOLFOX6 chemotherapy for patients with unresectable pseudomyxoma peritonei”.

They proposed that our study was a retrospective cohort study rather than a retrospective case series, and cited the description proposed by Dr. Dekker et al. [1]. From their distinguishing method between cohort studies and case series [1], our study meets the characteristic of cohort study: the patients were sampled on the basis of exposure, not a specific outcome, and followed over time to assess the occurrence of the outcomes. This description would be a nice candidate method to distinguish between cohort studies and case series, however, we do not think that this description has been completely regarded as a world-wide consensus.

For example, some researchers proposed that cohort studies differ from case series in that a comparison group is used for analysis of the treatment outcomes [2, 3]. In addition, the Strengthening the Reporting of Observational Studies in the Epidemiology (STROBE) recommendations refer to three main study designs including cohort, case–control, and cross-sectional studies, but do not specifically address case reports and case series [4]. The definition of case series has thus remained to be fully determined.

Taken together, at present, we consider that our study is not out of a case series. We completely agree with Dr. Luo and Dr. Li that the distinction between cohort studies and case series needs to be clarified in the future.
